# The impact of human population pressure on flying fox niches and the potential consequences for Hendra virus spillover

**DOI:** 10.1038/s41598-017-08065-z

**Published:** 2017-08-15

**Authors:** Michael G. Walsh, Anke Wiethoelter, M. A. Haseeb

**Affiliations:** 1 Marie Bashir Institute for Infectious Diseases and Biosecurity, Westmead Institute for Medical Research, University of Sydney, Westmead, New South Wales Australia; 2 Faculty of Veterinary and Agricultural Sciences, University of Melbourne, Melbourne, Victoria Australia; 3 Department of Epidemiology and Biostatistics, School of Public Health, State University of New York, Downstate Medical Center, Brooklyn, New York USA; 4 Departments of Cell Biology, Pathology and Medicine, College of Medicine, State University of New York, Downstate Medical Center, Brooklyn, New York USA

## Abstract

Hendra virus (HeV) is an emerging pathogen of concern in Australia given its ability to spillover from its reservoir host, pteropid bats, to horses and further on to humans, and the severe clinical presentation typical in these latter incidental hosts. Specific human pressures over recent decades, such as expanding human populations, urbanization, and forest fragmentation, may have altered the ecological niche of *Pteropus* species acting as natural HeV reservoirs and may modulate spillover risk. This study explored the influence of inter-decadal net human local migration between 1970 and 2000 on changes in the habitat suitability to *P. alecto* and *P. conspicillatus* from 1980 to 2015 in eastern Australia. These ecological niches were modeled using boosted regression trees and subsequently fitted, along with additional landscape factors, to HeV spillovers to explore the spatial dependency of this zoonosis. The spatial model showed that the ecological niche of these two flying fox species, the human footprint, and proximity to woody savanna were each strongly associated with HeV spillover and together explained most of the spatial dependency exhibited by this zoonosis. These findings reinforce the potential for anthropogenic pressures to shape the landscape epidemiology of HeV spillover.

## Introduction

Human pressures in sylvan landscapes can create circumstances favorable to the emergence of novel pathogens. In particular, the alteration of natural habitat often leads to new configurations of ecosystems and animal populations, which subsequently generate increased or unprecedented contact between human, domestic animal and wildlife communities^1–4^. Indirect as well as direct contact between humans and wildlife in such altered landscapes exposes the former to novel pathogens, which may present difficult public health challenges^3–6^. In recent years, bats in the family Pteropodidae have been identified as natural reservoirs of a number of emerging zoonotic viruses such as Nipah (NiV), Hendra (HeV), and Ebola. Their suitability as reservoirs has often been linked to unique characteristics, e.g. mammalian flight, adaptability to different food sources, population structure, longevity and immune function^7, 8^. Bats play critical roles in ecosystems of diverse geographic extent. More specifically, fruit bats of the genus *Pteropus*, also known as flying foxes, are essential pollinators and seed distributors^9, 10^ in tropical and subtropical forests of high biodiversity, and anthropogenic changes in their habitat exemplify the precarious balance between ecosystem integrity and human public health. NiV and HeV, for example, highlight the complex dynamics and novel exposures that attend virus emergence in altered ecosystems. While the landscape epidemiology and infection ecology remain incomplete for both of these henipaviruses, important strides have been made. Fragmented landscapes were associated with increased risk for NiV spillover in South and Southeast Asia^11^, with particularly strong evidence of proximity to disturbed forest landscape in Bangladesh^12, 13^. Climate has also been predicted to influence future spillover of both NiV and HeV, with warming temperatures increasing risk in many areas across Asia and Australia, respectively^3, 14^. In Australia, four species of flying foxes are native to the mainland: the black flying fox (*Pteropus alecto)*, the spectacled flying fox (*P. conspicillatus)*, the grey-headed flying fox *(P. poliocephalus)* and the little red flying fox *(P. scapulatus)*. The landscape epidemiology of HeV spillover has suggested that expanding suburban communities may draw foraging flying foxes from proximal forest ranges into the encroaching residential and community gardens^2, 15^ and thereby closer to horses. This may be particularly relevant for generalist pteropid foragers, such as *P. alecto* ^9^. Although exact HeV excretion and transmission routes between flying foxes and horses remain unknown, urine is considered to be the primary source of infection based on field investigations^16^. Additionally simulation models have indicated that HeV survival in the environment may be longer during colder periods, which corresponds to the observed seasonality of spillover and may further suggest a short window for potential horse exposure^17^. Evidence from several field surveys over the last decade have shown that *P. alecto* and *P. conspicillatus* shed infectious virus particles in urine whereas *P. poliocephalus* and *P. scapulatus* do not demonstrate viral shedding^16, 18–20^. As such, these data suggest that *P. alecto* and *P. conspicillatus* are the most likely natural reservoirs, while *P. scapulatus* and *P. poliocephalus* are incidental hosts. The investigation by Field *et al*.^20^, which examined urine samples from bat roosts from northern Queensland (QLD) to southern New South Wales (NSW), has been particularly important in showing that *P. alecto* is the likely primary natural reservoir across most of their range^16, 20^. Moreover, as these investigators have shown, the distribution of this flying fox is not uniform across the vast coastal corridor of eastern Australia. Nevertheless, the changing shape of this (and the other likely HeV reservoir, *P. conspicillatus*) species’ distribution in response to human population pressures over time remains unclear^15, 21^, as does the extent to which the possible expansion of the reservoir directly informs the spatial dependence of HeV spillover. The current study sought to explore whether the changing shape and expansion of the ecological niche of flying foxes between 1980 and 2015 may reflect decadal local human migration patterns and attendant environmental pressures. First, the ecological niche of *Pteropus* bats acting as HeV reservoirs in Australia was modeled per decade based on abiotic factors of climate, topography, and net internal human migration in the preceding decade. Second, HeV spillover incidents from 2000 to 2015 were fitted as a point process to these reservoir niche models to examine the association between a changing reservoir niche driven by human population pressure and HeV spillover risk, while simultaneously assessing the structure of unique land cover types, the human footprint, and the overall loss of vegetation. It was hypothesized that an inter-decadal expansion of the reservoir niche would be associated with a concurrent increasing linear trend in spillover risk.

## Methods

### Data sources

The records of documented *Pteropus* spp. sightings and specimens were obtained from the Global Biodiversity Information Facility (GBIF, http://www.gbif.org/). Between 1980 and 2015, there were 7126 geolocated field records of *Pteropus* spp. recorded within the spatial extent of NSW, QLD, and the Northern Territory (NT). Of this total, 1613 were *P. alecto*, 100 were *P. conspicillatus*, 1358 were *P. scapulatus*, and 4055 were *P. poliocephalus*. However, as described above, only the former two species demonstrate evidence of HeV shedding in urine and so *P. alecto* and *P. conspicillatus* alone were included in the sample (n = 1713) used to model the ecological niche of the reservoir host^16, 18–20^. No HeV spillovers have been reported in the NT to date. Nevertheless, the ecological niche of *P. alecto* demonstrated both wide range (during the earlier period) and substantive change over time. Since the purpose of this study was first to identify whether the reservoir niche was influenced by changing human populations, and then subsequently to examine whether the changing niche was associated with the spatial dependence of HeV, we included the NT to avoid biasing our results toward a strong spatial dependence of HeV on the reservoir niche.

A summary of all rasters described below, including source urls, is provided in the Supplemental Table. The WorldClim Global Climate database was the source of all climate data used in this investigation^22^. Mean temperature for the hottest and coldest quarters, and mean precipitation for the wettest and driest quarters from 1950 to 2000 were each extracted as 30 arc second resolution rasters^23^. Each pixel in these rasters represents the value of the measurement for that approximately 1 km^2^ area on the Earth’s surface.

Thirty arc-second rasters of net human migration between 1970 to 1980, 1980 to 1990, and 1990 to 2000, were obtained from the Socioeconomic Data and Applications Center (SEDAC), which is part of the National Aeronautics and Space Agency’s Earth Observing System Data and Information System^24^. The density was derived from the Global Rural-Urban Mapping Project estimates for the year 2000^25^. Net human migration for each of the three 10-year periods was measured as the net population flow into and out of each 1 km^2^ area and reflects changes due to both domestic and international population flux, rather than changes due to immigration. Positive values indicate a net gain in population, while negative values indicate a net loss^26, 27^.

The human footprint (HFP) was quantified using data also obtained from SEDAC^28^. The HFP was calculated in two stages. First, the human influence index (HII) was constructed. The HII measures the impact of human presence on the landscape as a function of eight domains: 1) population density, 2) proximity to railroads, 3) proximity to roads, 4) proximity to navigable rivers, 5) proximity to coastlines, 6) intensity of nighttime artificial light, 7) location in or outside delineated urban space, and 8) land cover. The domains are scored according to the level of human impact per geographic unit, whereby higher scores signify greater human influence. A composite index is then created by combining the eight individual domains. This composite ranges from 0, indicating an absence of human influence (i.e. a parcel of land unaltered by human activity), to 64, indicating maximal human influence in the landscape. The HII composite is subsequently normalized according to the 15 terrestrial biomes defined by the World Wildlife Fund to obtain the HFP. The normalization is represented as a ratio of the range of minimum and maximum HII in each biome to the range of minimum and maximum HII across all biomes, and is expressed as a percentage with a spatial resolution of approximately 1 km^2^ ^2, 29^.

The Moderate-resolution imaging spectroradiometer (MODIS)-based Maximum Green Vegetation Fraction (MGVF) was acquired from the United States Geologic Survey’s Land Cover Institute and used as a measure of vegetation cover^30^. At a resolution of 1 km^2^, two rasters recording the percentage of green vegetation cover per pixel in the years 2001 and 2010 were obtained and are a function of the normalized difference vegetation index^31^. The difference between the two rasters was calculated to determine the vegetation loss per 1 km^2^ between 2001 and 2010.

A 1 km^2^ raster of the distribution of 17 distinct land cover types based on the MODIS Land Cover Type (MCD12Q1) data product^32^ was obtained from the United States Geologic Survey’s Land Cover Institute. The land cover types represented between 2001 and 2010 were as follows: water, evergreen needle leaf forest, evergreen broadleaf forest, deciduous needle leaf forest, deciduous broadleaf forest, mixed forest, open shrubland, closed shrubland, woody savanna, savanna, grassland, permanent wetland, cropland, cropland/natural vegetation mosaic, urban/developed, sparse landscape, snow and ice, bare soil/sparse vegetation. Of the 17 land cover types, only 9 were substantively present in the states of NSW, QLD, and the NT so the analyses were limited to present land cover types: evergreen broadleaf forest, open shrubland, woody savanna, savanna, grassland, permanent wetland, cropland, cropland/natural vegetation mosaic, urban/developed. These 9 land cover types were extracted from this MODIS raster and new distance rasters created for each. The distance calculations were performed in the QGIS geographic information system (http://www.qgis.org/) using the proximity function. The result of this process was a separate raster of 1 km^2^ resolution for each of the 9 land cover types described above wherein the value of each pixel is the distance in meters between that pixel and the nearest pixel with the particular land cover feature present. This allows for modeling a spectrum of proximity to different land cover types in a given space rather than the more crude approach of simply designating the space as present or absent for specific land cover (see modeling description below).

Lists of HeV spillovers to horses were obtained from the Australian Veterinary Association (http://www.ava.com.au/hendra-virus#outbreaks) and the Government of Queensland’s Business and Industry Portal (https://www.business.qld.gov.au/industry/service-industries/veterinary-surgeons/guidelines-hendra/incident-summary). The data archives were further supplemented by searching the ProMED-mail database (http://www.promedmail.org/) and governmental websites for additional reports such as HeV bulletins and updates. Fifty-five of these spillover incidents with confirmed HeV cases were recorded between 1994 and 2015, with 52 cases occurring between 2000 and 2015. One spillover incident was reported with serological confirmation in a person in close contact with a HeV suspected but, due to a lack of suitable samples, unconfirmed horse case^33, 34^. The geographic coordinates for each spillover were obtained from Google Maps and cross-checked with Open Street Map as the centroid of the polygon of the local municipality in which the occurrence was reported.

### Statistical Methods

A boosted regression tree (BRT) modeling approach was employed in this study to model the ecological niche of the two HeV natural reservoir species, *P. alecto* and *P. conspicillatus*, at three different time points. Presence points were represented by the documented occurrences *P. alecto* and *P. conspicillatus*, in NSW, QLD, and the NT obtained from the GBIF database, with 251 observations taken from 1980 to 1989, 645 observations taken from 1990 to 1999, and 817 observations taken from 2000 to 2015. Pseudo-absence points are represented by a random sample of 250, 645, and 800 background points, respectively, in the same three states excluding the presence points from the geographical sampling frame. Reporting bias was assessed by additionally selecting sets of background points weighted by distance to roads from 1980 to 2010^35^ as a surrogate for pteropid observation accessibility^36^. There was no difference in the identified niches using the weighted and unweighted background and therefore the unweighted background points described above were used so that HFP, which was correlated with distance to roads, could be fitted with the flying fox niches in the subsequent spatially explicit models of HeV spillover (see model description below). Raster values for each landscape factor were then extracted at each presence and background point and the values concatenated in a data frame for analysis. Three distinct ecological niches were then modeled corresponding to the three periods of observation of the sampled bats: 1980–1989, 1990–1999, and 2000–2015.

The BRT models were used to predict HeV reservoir *Pteropus* spp. habitat suitability^37, 38^, and output maps of the predicted ecological niches for *P. alecto* and *P. conspicillatus* were created. The gradient boosted models used for these rasters included mean temperature during the warmest quarter, mean temperature during the coldest quarter, mean precipitation during the wettest quarter, mean precipitation during the driest quarter, altitude, and net human migration in the preceding decade (i.e. net migration from 1970–1980, 1980–1990, and 1990–2000, respectively) as predictors of flying fox habitat suitability at a 1 km^2^ resolution. Each of the three gradient boosted models specified 10000 regression trees, since the optimized models were estimated at 9981, 9985, and 9998 trees, for the 3 time periods, respectively, and were fitted with the shrinkage set to 0.001, which maximizes the learning rate for a large number of trees^37, 38^. Each model was fitted using 10-fold cross-validation and the area under the curve (AUC) was used to assess model performance. The model AUCs were corrected for potential spatial sorting bias with pairwise distance sampling^39^.

Kernel density estimation was used to estimate the density of HeV spillover incidents in eastern Australia. An isotropic Gaussian function was used for the kernel function, where the normal reference bandwidth was used as the default bandwidth^40^. The 52 HeV spillover incidents between 2000 and 2015 were modeled as a point process^41^. This is a spatially explicit approach, which investigates whether the distribution of spillovers are spatially dependent, and models the dependency as a function of specified landscape features. The point process was fitted separately to each decadal model of pteropid habitat suitability to determine how well the changing niche described the spatial dependence of spillover incidents between 2000 and 2015. First, a null model of complete spatial randomness was applied to the HeV outbreaks as a homogeneous Poisson process with conditional intensity,

1 $${\rm{\lambda }}({\rm{u}},{\rm{X}})={\rm{\beta }}$$

where u represents the set of locations corresponding to the HeV spillover points, X, and β is the intensity parameter. Under this model, the expected number of points (i.e. intensity) in any subregion of the geographic extent is simply proportional to the area of that subregion^41^.

Second, the fitted homogeneous Poisson process was compared to an inhomogeneous Poisson process model, which indicates a spatial dependency in the pattern of HeV spillover, and has conditional intensity,

2 $${\rm{\lambda }}({\rm{u}},{\rm{X}})={\rm{\beta }}({\rm{u}})$$

which shows the intensity as a function of the location, u, of the points. The inhomogeneous Poisson process proved to be a better fit (see Results), suggesting that the intensity was spatially dependent and, thus, the use of a spatially explicit model to describe HeV spillover dependency on the ecological niche of pteropid bats was deemed appropriate. One point process model was fitted to the pteropid niche from each of the three time periods (1980–1989, 1990–1999, 2000–2015), corresponding to three separate models. One period-specific raster describing these niches was included in each of three inhomogeneous point process models as a spatially explicit covariate with conditional intensity,

3 $${\rm{\lambda }}({\rm{u}},{\rm{X}})={\rm{\rho }}({\rm{Z}}({\rm{u}}))$$

where ρ is the function describing the association between the point intensity and the covariate Z at location u. Finally, a fourth model was built, which, in addition to the 2000 to 2015 pteropid niche, also incorporated the human footprint, vegetation loss between 2001 and 2010, and proximity to the 9 land cover types described above. Since the overall objective was to identify whether a changing reservoir niche was sufficient to explain the spatial dependency of HeV spillover, this fourth model was critical for evaluating the period-specific niches alone and in combination with other landscape features to identify the suite of features providing the best fit. The association between the intensity of HeV spillover and each covariate was assessed via the relative risks derived from the regression coefficients of the inhomogeneous Poisson model, and model fit was assessed using Akaike information criterion (AIC) and the spatial distribution of the smoothed residuals.

The R language was used for all analyses^42^. The gbm function in the gbm package was used to fit the gradient boosted models for the regression tree models^38, 43^, and the gbm.perf function used to identify the optimal number of trees. The kde.points function in the GISTools package was used to estimate the kernel function^44^. The ppm function in the spatstat package was used for the point process models^45, 46^.

## Results

**Figure 1:**
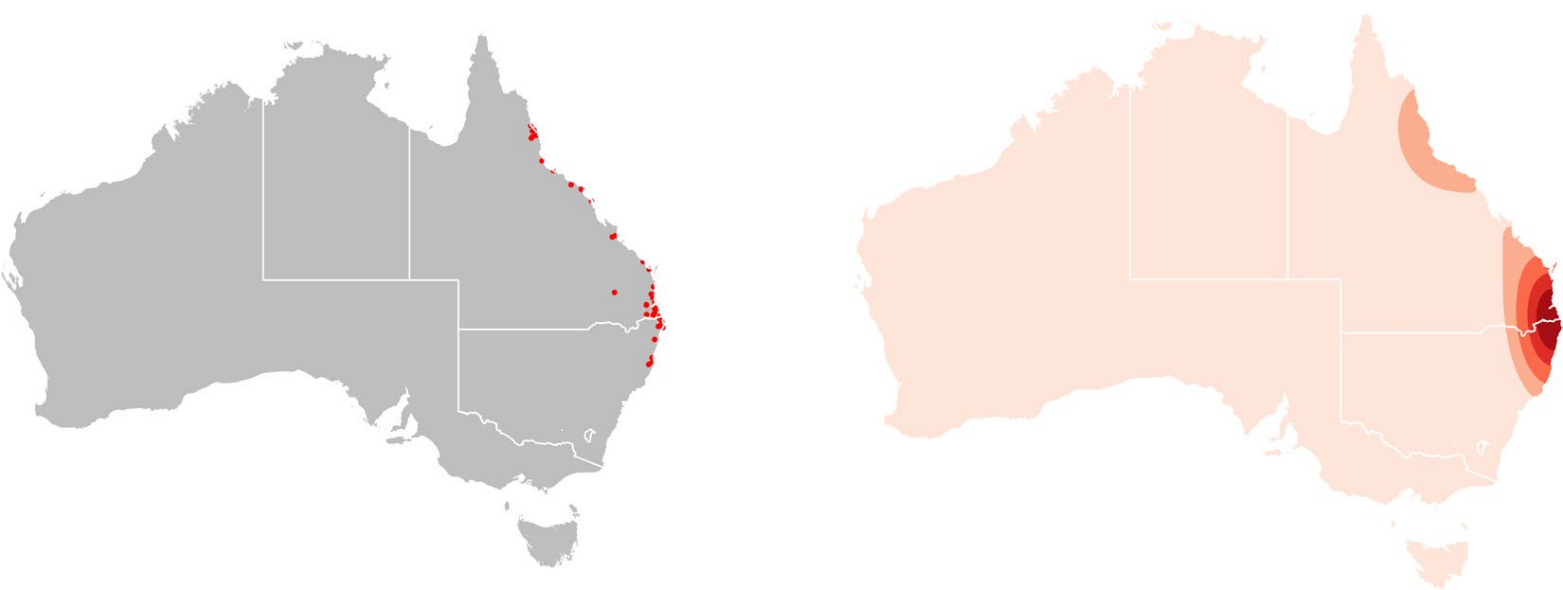
Hendra virus spillover. Left panel: Distribution of Hendra virus spillover incidents to horses in Queensland and New South Wales, Australia, between 2000 and 2015. Right panel: Kernel density estimate (KDE) of Hendra virus zoonotic transmission events. The KDE is mapped with Australian state boundaries. The white borders show the states used in the subsequent flying fox ecological niche analyses. All maps created in R (v. 3.3.1)^42^.

The distribution of the 52 HeV spillover incidents between 2000 and 2015 is presented in the left panel of Fig. 1, while the kernel density of these occurrences is presented in the right panel. These spillovers present a distinct corridor of occurrence along a narrow margin of eastern QLD and northeastern NSW and serve as a useful frame of reference for the investigation of the habitat suitability for the natural HeV reservoirs.

**Figure 2:**
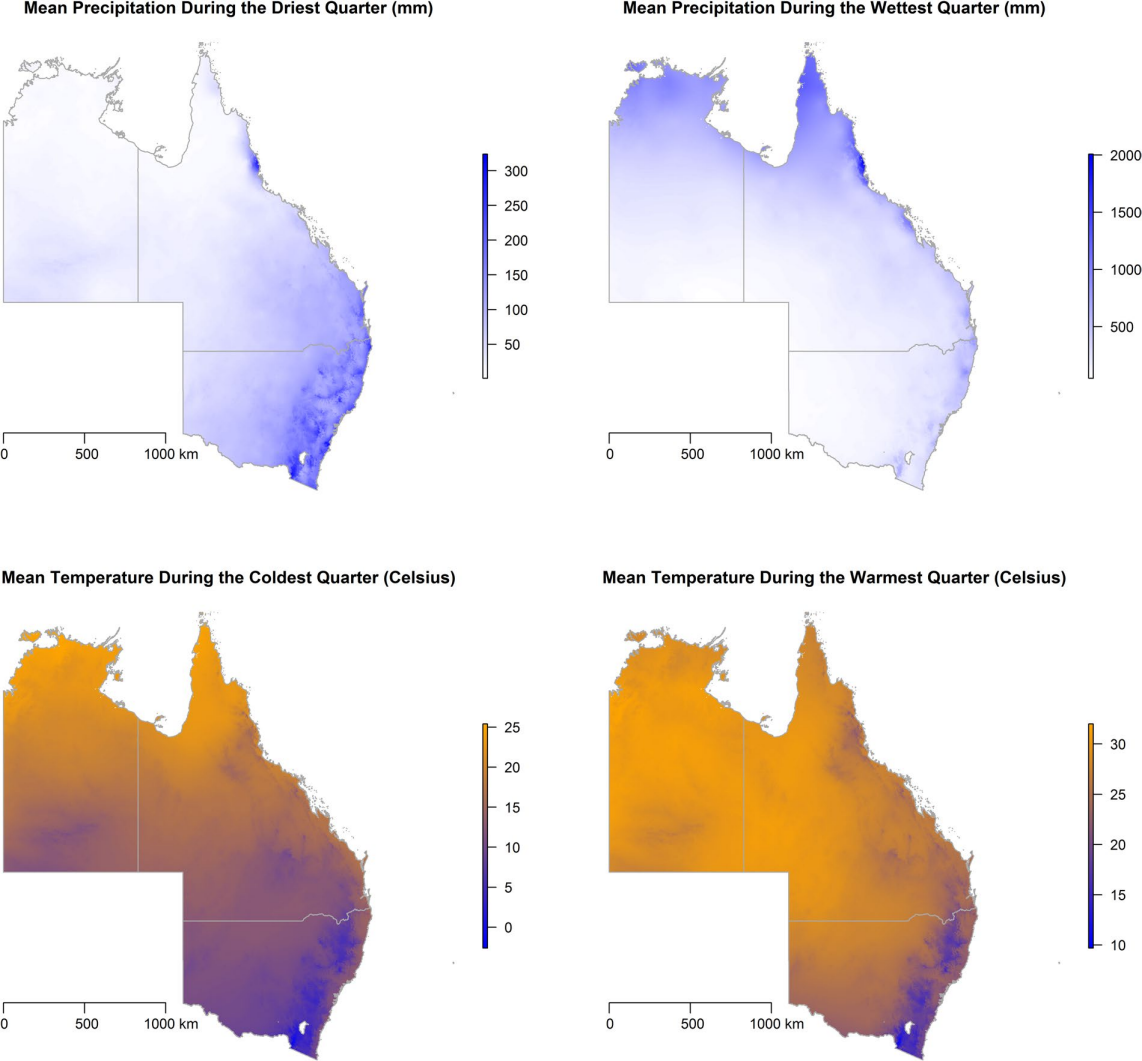
Climate distribution maps. The distribution of mean precipitation during the driest and wettest quarters and mean temperature during the coldest and warmest quarters. All maps created in R (v. 3.3.1)^42^.

**Figure 3:**
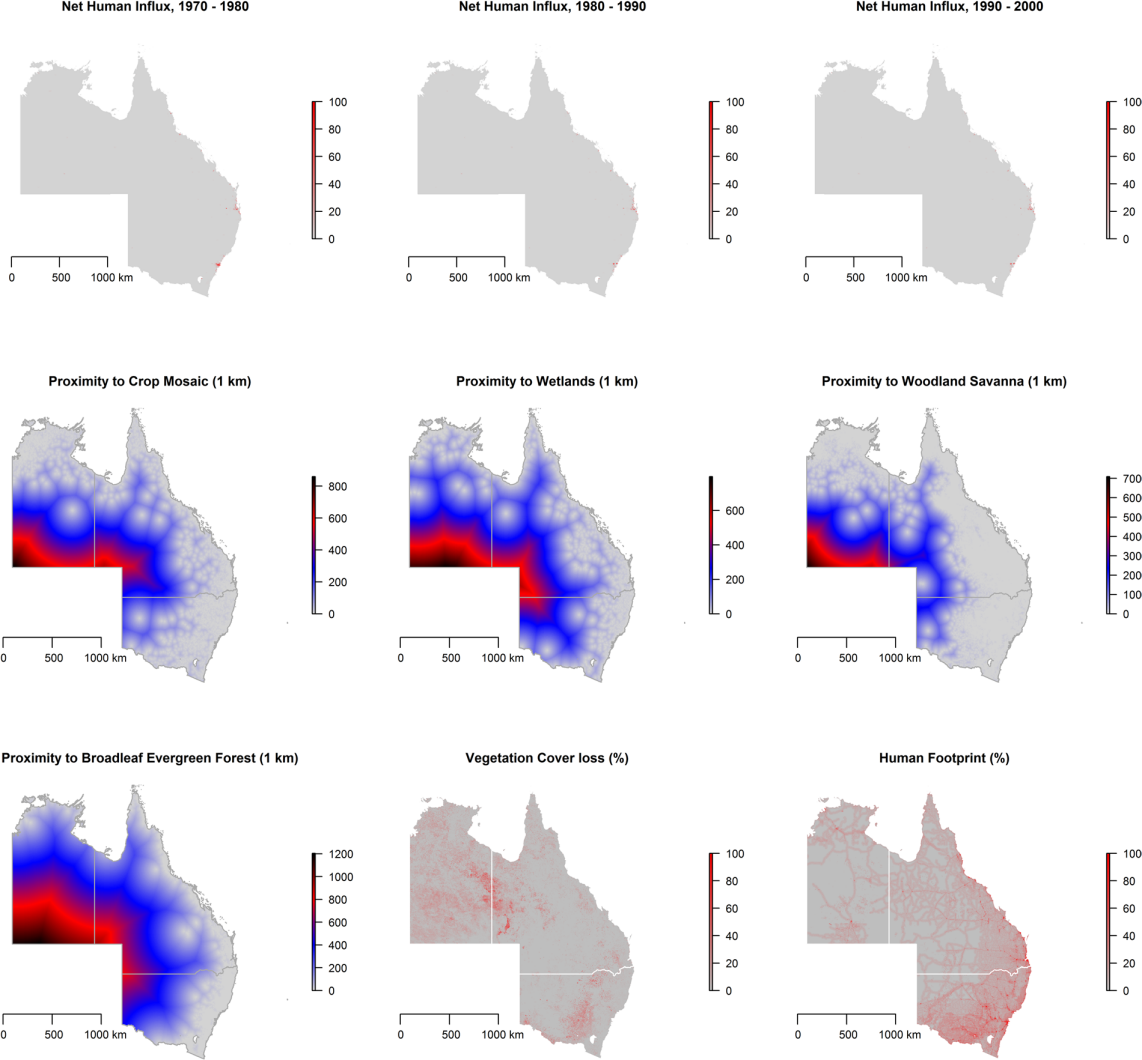
Landscape feature distribution maps. The first three maps in the top row represent the net human migration during the periods 1970–1980, 1980–1990, and 1990–2000, respectively. Each pixel in each of the subsequent four rasters represents the distance between that 1 km^2^ area and the nearest pixel containing that specific land cover feature. The last two rasters in the bottom row represent percent vegetation loss and the human footprint, respectively. All maps created in R (v. 3.3.1)^42^.

**Figure 4:**
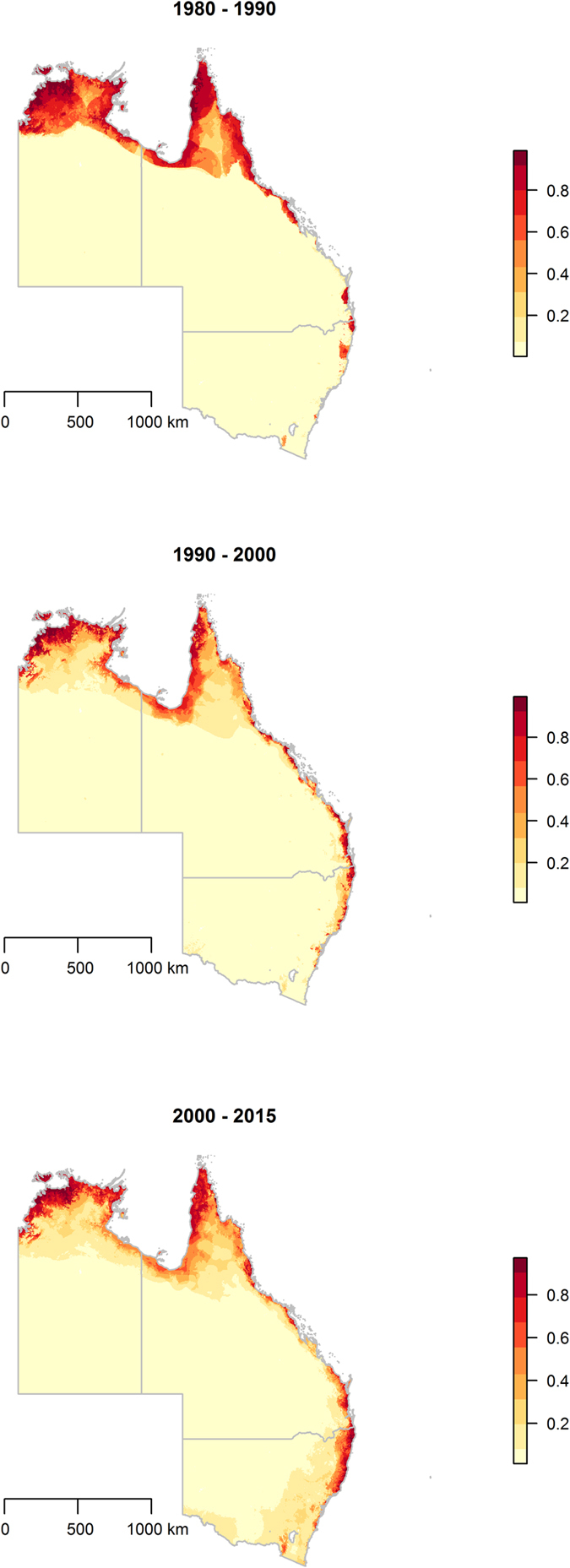
Changing ecological niche of Hendra virus reservoirs. These maps show the probability of landscape suitable to natural reservoirs of Hendra virus for each of three periods based on observations of *Pteropus alecto* and *P. conspicillatus* during 1980–1989, 1990–1999, and 2000–2015, respectively. The ecological niches were modeled using boosted regression trees. All maps created in R (v. 3.3.1)^42^.

The distributions of climate factors are presented in a series of maps in Fig. 2, while decadal human migration, proximity to land cover types, vegetation loss, and the human footprint are presented in Fig. 3. These maps allow for a visual descriptive analysis of each landscape factor under consideration in the modeling of the ecological niche of HeV reservoirs and spatial dependency of HeV spillover incidents. The maps in Fig. 4 show the predicted habitat suitability for *P. alecto* and *P. conspicillatus* for each of the three time periods based on the BRT-modeled ecological niches with model characteristics presented in Table 1. Precipitation during the wettest quarter and altitude were consistently the most influential predictors of the ecological niche of *P. alecto and P. conspicillatus* across the three decades with 83% and 7.2%, 43.4% and 40.9%, and 57% and 23.4% relative influence during the periods 1980–1989, 1990–1999, and 2000–2015, respectively. Net human migration was moderately influential during the first decade (4%), but became a much more substantive determinant of the changing ecological niche of pteropid bats during the second decade (16%). However, the effect of population flux on the evolving niche appears to have reached a threshold by the third time period as the relative influence was only 0.2% by 2000–2015. In addition, temperature during the coldest quarter and precipitation during the driest quarter demonstrated modest influence on the ecological niches across the three time periods. The predicted habitat suitability for HeV reservoir pteropids can be seen to expand geographically southward along the eastern coast of Australia from the earliest period in 1980–1989 to the latest in 2000–2015, with southeastern QLD and northeastern NSW exhibiting consistently high habitat suitability while advancing toward and beyond Sydney (Fig. 4). There was also a corridor along the northern coast of the NT that exhibited a high degree of predicted habitat suitability. Model evaluation demonstrated an improvement in model prediction from 83% to 88% AUC for this expanding ecological niche. Although model performance was somewhat attenuated for the last time period wherein population flux was less influential, these models showed generally good predictive performance overall.

**Table 1:** Description of the ecologic niche models for Hendra reservoir *Pteropus* species for each decade 1980–1989, 1990–1999, and 2000–2015, respectively.

Predicted pteropid SDM	AUC	Rank 1 (%)	Rank 2 (%)	Rank 3 (%)	Rank 4 (%)	Rank 5 (%)
1980–1989	83%	Precip, wettest qtr (83.0)	Altitude (7.2%)	Migration 1970–1980 (4.0)	Temp, coldest qtr (3.4)	Precip, driest qtr (1.3)
1990–1999	88%	Altitude (43.4)	Precip, wettest qtr (40.9)	Migration 1980–1990 (16.0)	Temp, coldest qtr (3.9)	Precip, driest qtr (1.8)
2000–2015	80%	Precip, wettest qtr (57.0)	Altitude (23.4)	Precip, driest qtr (9.0)	Temp, warmest qtr (5.7)	Temp, coldest qtr (4.6)

For each model the area under the curve (AUC) is presented to describe model performance, and the ranking of the top five landscape features defining the ecologic niche are listed with their respective relative influences reported as a percentage in parentheses. The rank order is determined by the magnitude of the relative influence. Precipitation (Precip) during the driest and wettest quarters (qtr), and temperature (Temp) during the warmest and coldest quarters represent the mean measurements during these quarters over the period 1950 to 2000. Migration represents the net human migration per 1 km^2^ during the previous decade.

**Figure 5:**
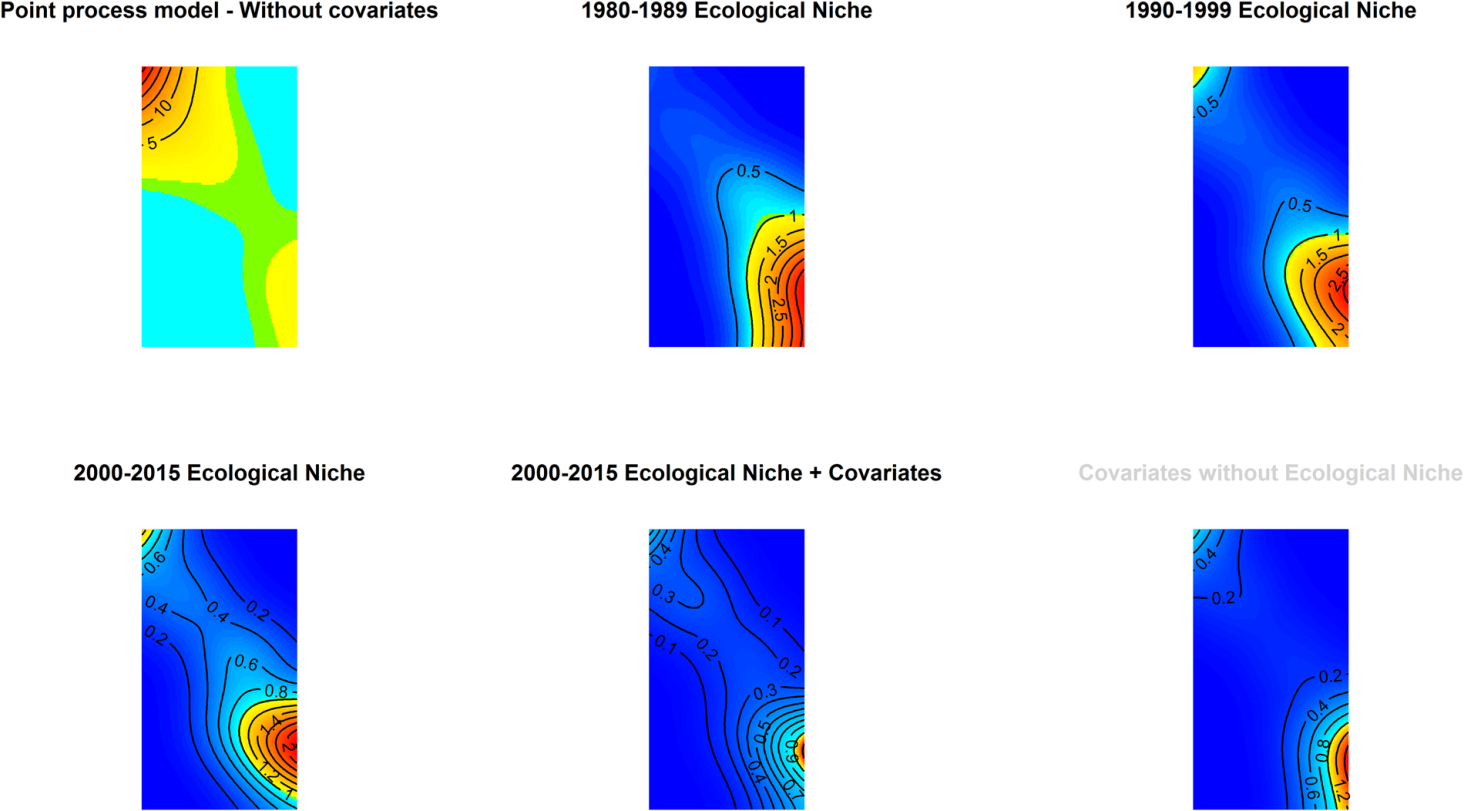
Smoothed residual plots. These plots demonstrate the degree of spatial autocorrelation in the inhomogeneous models of Hendra virus spillover incidents with each period-specific ecological niche of Hendra virus reservoirs (*Pteropus alecto* and *P. conspicillatus*) both unadjusted and adjusted for the human footprint, vegetation loss, and the woody savanna.

The simple and multivariable inhomogeneous Poisson process models are presented in Tables 2 and 3, with relative risks representing the unadjusted and adjusted measures of association, respectively, between HeV spillover and ecological niches of HeV reservoirs by decade and other landscape factors. Fitting spillover incidents to the predicted reservoir ecological niches showed that for each decade under consideration the proximity to suitable reservoir habitat was strongly associated with the spillover incidents that occurred between 2000 and 2015 (Table 2). Moreover, as the ecological niche expanded from the periods 1980–1989 to 1990–1999 the association between proximity to suitable habitat and spillover increased by three-fold (1980–1989: RR = 51.0, 95% C.I. 25.7–100.9; 1990–1999: RR = 174.1, 95% C.I. 72.9–421.8), and increased further still as habitat suitability increased from 2000–2015 (RR = 196.4, 95% C.I.78.6–490.7). While the ecological niche of HeV reservoirs during each decade was strongly associated with subsequent HeV spillover incidents, and while there was a clear increasing trend in spillover risk as the niche expanded during the first two decades under study, none of the decade-specific niches were necessarily a good fit to the spillover incident data when considered in isolation as demonstrated by the spatial autocorrelation of the residuals. Figure 5 presents the spatially smoothed residuals for each model. The first field depicts the spatial dependence present in a model with no predictors, i.e. the spillover incidents are fitted to the spatial coordinates alone and reveal their strong spatial dependency. The subsequent three fields represent the models fitting the reservoir ecological niches for each time period. While each of the three models showed that the spatial dependence was considerably reduced when the spillovers were fitted to these niches, there nevertheless remained substantive unexplained spatial dependence. This suggests that an expanding reservoir niche, while an important and probably necessary antecedent to HeV spillover, is insufficient to explain the spatial pattern in spillover incidents that emerged from 2000 to 2015 and thus requires an additional fourth model. The fourth model (Table 3) fitted the same spillover incidents to the reservoir ecological niche during the period 2000 to 2015 as well as the human footprint, vegetation loss, and land cover type over the same time period. The reservoir niche remained strongly associated with spillover risk (RR = 74.1, 95% C.I. 23.7–231.9), though attenuated, once adjusted for the other landscape factors. The human footprint (RR = 1.04, 95% C.I. 1.03–1.05) and proximity to woody savanna (RR = 1.63, 95% C.I. 1.13–2.34) were also significantly associated with spillover risk, with each 1% increase in the footprint corresponding to a 4% increase in risk and each 100 meter increase in proximity to woody savanna corresponding to a 63% increase in risk, respectively. Most importantly, the inclusion of these additional landscape factors represented the best overall fit to the HeV spillover data as can be seen by the reduced spatial autocorrelation of the residuals in the second to last panel of the Fig. 5. The smoothed residual field based on the full model with the 2000–2015 ecological niche plus additional landscape factors (2^nd^ panel in the 2^nd^ row) showed that the intensity was generally uniformly estimated by this model across most of the spatial extent, thus demonstrating a reasonable fit to the HeV spillover data. The last panel presented in Fig. 5 reflects a model with the additional landscapes factors included, but excluding the ecological niche of HeV reservoirs. This model was included to demonstrate that the best fitting model as measured by maximally reduced spatial autocorrelation was not entirely due to the inclusion of these additional landscape factors.

**Table 2:** Unadjusted relative risks and 95% confidence intervals for the associations between Hendra virus spillover incidents and the period-specific ecological niches of natural Hendra virus reservoirs *(P. alecto* and *P. conspicillatus*) (Model 1: 1980–1989 niche, Model 2: 1990–1999 niche, Model 3: 2000–2015 niche).

Period-specific HeV reservoir ecological niche	Relative Risk	95% C.I.
Model 1: 1980–1989 Ecological niche of *Pteropus alecto* and *P. conspicillatus* (%)	51.0	25.7–100.9
Model 2: 1990–1999 Ecological niche of *Pteropus alecto* and *P. conspicillatus* (%)	174.1	72.9–421.8
Model 3: 2000–2015 Ecological niche of *Pteropus alecto* and *P. conspicillatus* (%)	196.4	78.6–490.7

These measures of association are derived from an inhomogeneous Poisson model of the point process of spillovers.

**Table 3:** Adjusted relative risks and 95% confidence intervals for the associations between Hendra virus spillover incidents and each landscape feature.

Landscape factor	Relative Risk	95% C.I.
2000–2015 Ecological niche of *Pteropus alecto* and *P. conspicillatus* (%)	74.1	23.7–231.9
Human footprint (%)	1.04	1.03–1.05
Maximum green vegetation fraction loss (%)	0.81	0.65–1.02
Proximity to woodland savanna (100 m)	1.63	1.13–2.34

These measures of association are derived from an inhomogeneous Poisson model of the point process of spillovers wherein each landscape factor is adjusted for all others.

## Discussion

This investigation explored the expanding ecological niche of the HeV reservoirs, *P. alecto* and *P. conspicillatus*, between 1980 and 2015 and the influence of internal human population pressures on the expansion and changing shape of this niche. While climate and topography consistently delineated the reservoir habitat suitability across the three decades under study, an increase in the net local influx of the human population from 1980 to 1990 was also influential to the emergent niche during 1990 to 2000. The spatial dependence of HeV spillovers on the modeled niches was marked for each of the three time periods. Moreover, an increasing trend in spillover risk was apparent for each period of ecological niche expansion between 1980–1989, 1990–1999, and 2000–2015. This corresponds with the history of HeV spillover incidents in Australia. First discovered in 1994 in an outbreak in a racing stable in Hendra, a suburb of Brisbane, QLD, this newly emerging infectious disease made several further sporadic occurrences between 1994 and 2010 until in 2011 an unprecedented number of 18 distinct spillovers more than doubled the number of known incidents^47^. Since then new spillover incidents have been detected on a yearly basis. The shared history between HeV spillover and the ecological niche of flying foxes notwithstanding, reservoir habitat suitability alone was insufficient to describe the spatial dependence of HeV spillover. The human footprint, proximity to woody savanna, and vegetation loss were additional components of the landscape required to adequately describe the spatial dependence of spillover across eastern Australia.

While *P. alecto*, *P. scapulatus*, *P. poliocephalus*, and *P. conspicillatus* are all susceptible to HeV infection, only *P. alecto* and *P. conspicillatus* appear to be natural reservoirs^16, 18–20, 48, 49^ and thus the distribution of these two species, realized and potential, were the focus of the current investigation. This study is not the first to examine the potential expansion of flying fox range in eastern Australia. A previous study found a 5-fold increase of *P. alecto* in its southern range over 10-year increments between 1950 and 2000^15^. Moreover, this group found that the expansion did not correspond to changes in climate, but must be consequent to other, as yet unknown, factors operating in the landscape. Specific processes of urbanization may encapsulate the relevant factors that draw flying foxes into anthropogenic landscapes. For example, loss of natural habitat typically attends urbanization and increasing human population pressure. As flying fox habitat is lost as a result, it may be supplanted with more readily available food sources in the form of urban gardens, or peri-urban horticulturally mixed landscapes^2, 50^. Evidence from telemetric data suggests that as anthropogenic change alters natural pteropid habitat, *P. alecto* is showing a preference for exotic food sources in close proximity to human residence^51^, or urban parks and gardens^9^. Similar findings have been demonstrated in the other natural reservoir species of HeV, *P. conspicillatus* ^21^. In addition to potentially expanding and reshaping the habitat suitability of flying foxes, urbanization may also lead to changes in the connectivity among and between populations, whereby increasingly fragmented landscapes lead to increasingly fragmented bat metapopulations^2^. This phenomenon is particularly relevant to HeV spillover. Metapopulation structure among *Pteropus* species has been suggested as a critical driver of HeV infection dynamics in flying foxes; loss of connectivity between populations, resulting from reduced migration within the metapopulation, was associated with increased size of HeV epizootics in *Pteropus* spp.^50^. As such, anthropogenic fragmentation of *Pteropus* habitat would be expected to exert pressures on bat social structure and movement, which in turn may facilitate HeV spillover^52^. For example, data has shown that decreased connectivity between bat populations leads to diminished enzootic transmission across the larger metapopulation, thus increasing overall susceptibility among the reservoir over time. Subsequently, when HeV is reintroduced to larger communities of immunologically naïve flying foxes, this results in more intense epizootics, which have greater potential to spillover to novel hosts^50^. In addition to affecting metapopulation partition in peri-urban space, anthropogenic ecotones may also act as bat community “sinks” because of the increased availability of food sources, as previously described above^9^.

Precipitation and altitude demonstrated consistently strong influence on the ecological niche of these reservoir flying foxes. This is not surprising since climate in general, and precipitation in particular, is known to influence the flowering of Eucalypts, a group of plants that includes preferred foraging tree species for flying foxes in Australia^53^. Moreover, the influence of Eucalypts cycling in response to seasonal climatic variation, has been shown to drive flying fox population flux in QLD^54^. In addition, another recent study modeled the ecological niche of all four Australian flying fox species as a function of a larger suite of climatic variables, and identified niches for *P. alecto* and *P. conspicillatus* very similar to those presented here. However, the former study did not evaluate the decadal changes in these niches in response to human population flux that we present in the current study, although they did identify an increased risk in HeV spillover associated with niche proximity^55^. Altitude has not previously been shown as an important abiotic factor delineating the ecological niche of Australian flying foxes, however this feature is likely a proxy for the availability of preferred foraging habitat, which tends to be closer to the coast and, thus, low-lying.

When fitted to HeV spillovers, the ecological niche models presented here were strongly associated with risk. While this corroborates the findings on urbanization described above, the model fit remained inadequate (as evidenced by the persistent spatial autocorrelation in the residuals) until additional landscape features were included in the model, which also agrees with previous results on urbanization and both bat distribution and HeV spillover. The influence of the human footprint directly on spillover reflects much of the discussion already elucidated with respect to its modulation of the distribution of flying foxes insofar as the anthropogenic alteration of the landscape often creates novel habitat that is favorable to HeV reservoir species^2, 21, 51, 56^, but which may simultaneously reduce metapopulation connectivity, diminish HeV circulation, and inhibit population immunity^50, 52^.

The relevance of human influence to HeV spillover dynamics along Australia’s east coast is also important for risk communication. Media coverage of HeV has emphasized that flying foxes, as the reservoirs, constitute a threat, leading to calls for either eradication or dispersal of flying foxes in urban landscapes by members of the public^57^. Moreover, qualtitative research with horse owners revealed that some try to deter bats with noise, smoke or shooting rather than implementing recommended risk mitigation measures such as vaccinating horses, covering feed and water or stabling horses over night^58^. Thus, incorporating the potential impact of anthropogenic changes into broader HeV risk narratives might facilitate the uptake of mitigation measures focused on horse and property management. Although Edson *et al*. found no association between increased HeV excretion and flying fox dispersal^59^, such activities might still impact negatively on metapopulation connectivity as well as individual animal welfare.

In addition to suitable pteropid habitat and an increasing human footprint, proximity to woody savanna was also associated with HeV spillover. Of all nine natural land cover types considered in eastern Australia, woody savanna demonstrated the strongest association and was the single land cover type selected in the best fitting model. This landscape feature has not previously been reported in association with HeV spillover risk, but is nevertheless intuitive given that this land cover type is a natural ecotone that may yield more easily than other land cover types to anthropogenic manipulation for crop development and/or pasture grazing, thus transitioning from natural to artificial ecotone and subsequently favorable pteropid habitat^60^. Nevertheless, while an interesting finding, its interpretation is limited given that our land cover data do not demarcate distinct tree species and therefore do not allow for the pairing of flying fox observations with known preferred roosting and feeding habitat. However, another study examining flying fox densities associated with individual roosts and food availability in eucalypt forests found that smaller, sedentary flying fox communities were associated with more spillover incidents than larger, actively migrating communities^54^. This is in agreement with the current investigation but provides greater hyperlocal context based on observed flying fox use of the landscape.

Our findings are also in agreement with two extensive longitudinal studies with respect to proximity to flying foxes. One investigation examined risk factors for HeV spillover between 1994–2010^61^. This study found that postal codes with pteropid roosts were forty times more likely to experience HeV spillover compared to those that did not. Similarly, a later study covering the period 1994–2012, identified proximity to flying foxes to be the most important predictor of spillover, independent of climate, vegetation cover, and horse density^62^. Interestingly, vegetation cover was no longer associated with HeV spillover in either of these studies after accounting for proximity to flying foxes^61, 62^. The latter result is concordant with our own finding that vegetation loss was not significant in the full spatial model, however it was discordant with our finding of the concomitant influence of woody savanna. The discrepancy could be due to 1) the previous study’s use of geolocated flying fox roosts in contrast to the reservoir ecological niche used in the current study, or 2) the use of more type-specific land cover assessed in the current study, or 3) a combination of both methodological differences.

The findings from the current study must be interpreted with caution due to some important limitations that warrant further discussion. First, temperature and precipitation were measured as single composites over the period 1950 to 2000. Therefore, while the spatial resolution of these measurements was high (~1 km^2^ rasters), the analysis was simultaneously constrained by coarse temporal resolution, given that the climate rasters were averaged over a 50 year time span. Nevertheless, it is felt that these measures are indicative of the general temperature and precipitation of the regions represented, and thus provide a reasonable and robust approach to assessing background climate. Second, horse density data were unavailable for the complete extent of eastern Australia at the necessary resolution for analysis in this investigation. The lack of horse density data is an important limitation to any attempt to model HeV spillover, as horses are the key amplifying hosts of HeV. However, the primary aim of this study was not to model HeV spillover, but rather to model the ecological niche of HeV reservoirs (flying foxes) and then subsequently fit the modeled niche to spillover incidents to determine the niche’s degree of fit. Further, horse density was not identified as a significant risk factor in previous studies^61, 62^. As such, we feel that the lack of horse data does not preclude all utility of the niche modeling exercise. It does, however, limit the extent to which we can conclude that the modeled niche predicts HeV spillover. The latter would require validation with horse density data generated at sufficiently fine-scaled resolution at the full extent currently under study, which does not exist at present. This limitation notwithstanding, the spatial model used in this investigation did prove to be a good fit to the data, demonstrating very little residual spatial autocorrelation in a point process of spillovers that was otherwise highly spatially dependent. Third, the number of occurrences of documented HeV spillovers between 2000 and 2015 is relatively small, which obviously limits the sample size available for analysis. Therefore, with only 52 occurrences in this subset, this collection of reported incidents may not be representative of the total incidents occurring in eastern Australia. Although heightened awareness of veterinarians and owners has led to increased HeV surveillance in horses in recent years, some level of under-reporting cannot be excluded due to the variety of clinical presentations of equine HeV infections^63^. Nevertheless, the current analysis has taken care to validate the sparse data by 1) demonstrating a valid point process for modeling spatial dependence, and 2) carefully assessing model fit as reflected in the spatial autocorrelation of the residuals. Fourth, vegetation loss, the human footprint, and land cover types were only assessed during the period 2000 to 2010. The former represented the difference between the start and end of the decade while the latter two represented the average across the decade. Therefore, it was not possible, as it was in assessing the influence of net human migration on the ecological niche of fly foxes, to assess earlier changes in these landscape features, whose origins undoubtedly lie more temporally distal to HeV spillovers beginning in 2000. In summary of the limitations, these data, and specifically the comparison of the modeled niche with spillovers, are not intended to posit a definitive understanding of HeV spillover incidents. This investigation sought primarily to identify whether evolving human population pressures were associated with distinct changes in the ecological niche of the natural reservoir of HeV, and secondarily to examine how well this niche described the spatial structure of HeV spillovers. We do not assert a direct causal interpretation of the associations between spillover and landscape factors as derived from the inhomogeneous Poisson model. The broad analytic scale of the point process of HeV spillovers is the primary limiting factor to such an interpretation because these incidents tend to be highly localized. The associations may suggest broad spatial patterns in the co-occurrence of spillover with the human footprint, land cover, and the ecological niche of flying foxes, but they do not define causal relationships. More definitive causal inference will require direct measurement of animals and humans in the landscapes where HeV spillovers are occurring and where they are not occurring, preferably at high spatial resolution. This will require extensive field investigations incorporating detailed habitat description at a much finer scale across a far wider geographic extent, and agricultural and animal husbandry practices that shape the human influence on the landscape and which bring novel hosts into contact with reservoir species.

In conclusion, this study identified a change in the ecological niche of two flying fox species following net increases in human population in-flow at a scale of 1 km^2^, particularly during the period 1990–1999. This changing geometry of suitable flying fox habitat was associated with subsequent HeV spillovers and, when considered together with the human footprint and land cover, proved effective in explaining the spatial dependency of these events. These results help to extend the landscape epidemiology and infection ecology of HeV spillover incidents, and may suggest intervention strategies simultaneously beneficial to public health and bat ecology.

## Electronic supplementary material

Supplementary information

## References

[1] Estrada-Peña A, Ostfeld RS, Peterson AT, Poulin R, de la Fuente J (2014). Effects of environmental change on zoonotic disease risk: an ecological primer. Trends Parasitol..

[2] Plowright, R. K. *et al*. Ecological dynamics of emerging bat virus spillover. *Proc. Biol. Sci*. **282** (2015). 10.1098/rspb.2014.2124 PMC4262174 25392474

[3] Daszak P (2013). Interdisciplinary approaches to understanding disease emergence: the past, present, and future drivers of Nipah virus emergence. Proc. Natl. Acad. Sci. USA.

[4] Daszak P, Cunningham AA, Hyatt AD (2000). Emerging infectious diseases of wildlife–threats to biodiversity and human health. Science.

[5] Wolfe ND, Dunavan CP, Diamond J (2007). Origins of major human infectious diseases. Nature.

[6] Woolhouse, M. E. J. & Gowtage-sequeria, S. Host. Range and Emerging and Reemerging Pathogens. **11** (2005). 10.3201/eid1112.050997 PMC3367654 16485468

[7] Wang LF, Walker PJ, Poon LLM (2011). Mass extinctions, biodiversity and mitochondrial function: Are bats ‘special’ as reservoirs for emerging viruses?. Curr. Opin. Virol..

[8] Calisher CH, Childs JE, Field HE, Holmes KV, Schountz T (2006). Bats: Important reservoir hosts of emerging viruses. Clin. Microbiol. Rev..

[9] Markus N, Hall L (2004). Foraging behaviour of the black flying fox (Pteropus alecto) in the urban landscape of Brisbane. Wildl. Res..

[10] Parsons JG (2006). Dietary variation in spectacled flying foxes (Pteropus conspicillatus) of the Australian Wet Tropics. Aust. J. Zool..

[11] Walsh MG (2015). Mapping the risk of Nipah virus spillover into human populations in South and Southeast Asia. Trans. R. Soc. Trop. Med. Hyg..

[12] Hahn MB (2014). The role of landscape composition and configuration on Pteropus giganteus roosting ecology and Nipah virus spillover risk in Bangladesh. Am. J. Trop. Med. Hyg..

[13] Hahn MB (2014). Roosting behaviour and habitat selection of Pteropus giganteus reveals potential links to Nipah virus epidemiology. J. Appl. Ecol..

[14] Breed AC (2013). The distribution of henipaviruses in Southeast Asia and Australasia: is Wallace’s line a barrier to Nipah virus?. PLoS One.

[15] Roberts BJ, Catterall CP, Eby P, Kanowski J (2012). Latitudinal range shifts in Australian flying-foxes: A re-evaluation. Austral Ecol..

[16] Edson D (2015). Routes of Hendra Virus Excretion in Naturally-Infected Flying-Foxes: Implications for Viral Transmission and Spillover Risk. PLoS One.

[17] Martin G (2015). Hendra virus survival does not explain spillover patterns and implicates relatively direct transmission routes from flying foxes to horses. J. Gen. Virol..

[18] Burroughs AL (2016). Hendra Virus Infection Dynamics in the Grey-Headed Flying Fox (Pteropus poliocephalus) at the Southern-Most Extent of Its Range: Further Evidence This Species Does Not Readily Transmit the Virus to Horses. PLoS One.

[19] Goldspink LK (2015). Natural Hendra Virus Infection in Flying-Foxes - Tissue Tropism and Risk Factors. PLoS One.

[20] Field H (2015). Spatiotemporal Aspects of Hendra Virus Infection in Pteropid Bats (Flying-Foxes) in Eastern Australia. PLoS One.

[21] Tait J, Perotto-Baldivieso HL, McKeown A, Westcott DA (2014). Are flying-foxes coming to town? Urbanisation of the spectacled flying-fox (Pteropus conspicillatus) in Australia. PLoS One.

[22] WorldClim - Global Climate. Data for current conditions (~1950–2000) | WorldClim - Global Climate Data. *WorldClim - Global Climate Data* at http://www.worldclim.org/current.

[23] Hijmans RJ, Cameron SE, Parra JL, Jones PG, Jarvis A (2005). Very high resolution interpolated climate surfaces for global land areas. Int. J. Climatol..

[24] Center for International Earth Science Information Network - CIESIN - Columbia University, International Food Policy Research Institute - IFPRI, The World Bank, and C. I. de A. T.-C. Population Density Grid, v1: Global Rural-Urban Mapping Project (GRUMP), v1 | SEDAC. Palisades, NY: NASA Socioeconomic Data and Applications Center (SEDAC) at http://sedac.ciesin.columbia.edu/data/set/grump-v1-population-density.

[25] Balk DL (2006). Determining global population distribution: methods, applications and data. Adv. Parasitol..

[26] De Sherbinin A (2012). Migration and risk: net migration in marginal ecosystems and hazardous areas. Environ. Res. Lett..

[27] De Sherbinin, A. *et al*. Global Estimated Net Migration Grids by Decade: 1970–2000. NASA Socioeconomic Data and Applications Center (SEDAC) at http://sedac.ciesin.columbia.edu/data/set/popdynamics-global-est-net-migration-grids-1970-2000 (2015).

[28] Sanderson EW (2002). The Human Footprint and the Last of the Wild. Bioscience.

[29] Socioeconomic Data and Applications Center | SEDAC. Methods » Last of the Wild, v2 | SEDAC. at http://sedac.ciesin.columbia.edu/data/collection/wildareas-v2/methods.

[30] The USGS Land Cover Institute. MODIS-based Maximum Green Vegetation Fraction. At http://landcover.usgs.gov/green_veg.php.

[31] Broxton, P. D., Zeng, X., Scheftic, W. & Troch, P. A. A MODIS-Based Global 1-km Maximum Green Vegetation Fraction Dataset. http://journals.ametsoc.org/doi/abs/ doi:10.1175/JAMC-D-13-0356.1 (2014).

[32] Broxton PD, Zeng X, Sulla-Menashe D, Troch PA (2014). A global land cover climatology using MODIS data. J. Appl. Meteorol. Climatol..

[33] Field HE (2007). Epidemiological perspectives on Hendra virus infection in horses and flying foxes. Aust. Vet. J..

[34] Hanna JN (2006). Hendra virus infection in a veterinarian. Med. J. Aust..

[35] Center for International Earth Science Information Network - CIESIN - Columbia University, and I. T. O. S. *Global Roads Open Access Data Set, Version 1 (gROADSv1)*. At doi:10.7927/H4VD6WCT (2013).

[36] Phillips, S. J. *et al*. Sample selection bias and presence-only distribution models: implications for background and pseudo-absence data Reference Sample selection bias and presence-only distribution models: implications for background and pseudo-absence data. **19**, 181–197 (2009). 10.1890/07-2153.1 19323182

[37] Elith J, Leathwick JR, Hastie T (2008). A working guide to boosted regression trees. J. Anim. Ecol..

[38] Friedman J (2001). Greedy Function Approximation: A Gradient Boosting Machine. Ann. Stat..

[39] Hijmans, R. J. & Hall, W. Cross-validation of species distribution models: removing spatial sorting bias and calibration with a null model Published by: Ecological Society of America Stable http://www.jstor.org/stable/23143954 REFERENCES Linked references are available on. *Ecology* **93**, 679–688 (2016). 10.1890/11-0826.1 22624221

[40] Venables, W. N. & Ripley, B. D. Modern Applied Statistics with S. at http://books.google.com/books?hl=en&lr=&id=974c4vKurNkC&pgis=1 (Springer, 2002).

[41] Baddeley, A. & Turner, R. Practical Maximum Pseudolikelihood for Spatial Point Patterns (with Discussion). Aust. html_ent glyph=‘@amp;’ ascii=‘&’/ *New Zeal. J. Stat*. **42**, 283–322 (2000).

[42] Team, R. C. R: A language and environment for statistical computing. At https://www.r-project.org/ (2016).

[43] Ridgeway, G. CRAN - Package gbm. at http://cran.r-project.org/web/packages/gbm/index.html.

[44] Brunsdon, C. & Chen, H. Documentation for package ‘GISTools’ version 0.7-1. at http://127.0.0.1:23595/library/GISTools/html/00Index.html (2012).

[45] Baddeley, A. & Turner, R. Spatstat: An R Package for Analyzing Spatial Point Patterns. *J. Stat. Softw*. **12**(6) at http://www.jstatsoft.org/v12/i06/ (2005).

[46] Baddeley, A., Rubak, E. & Turner, R. *Spatial Point Patterns: Methodology and Applications with R*. **11** (CRC Press, 2015).

[47] Field, H. E. Hendra virus ecology and transmission. *Curr. Opin. Virol*. **16**, 120–125 (2016). 10.1016/j.coviro.2016.02.004 26978066

[48] Halpin K, Young PL, Field HE, Mackenzie JS (2000). Isolation of Hendra virus from pteropid bats: a natural reservoir of Hendra virus. J. Gen. Virol..

[49] Young, P. L. *et al*. Serologic evidence for the presence in Pteropus bats of a paramyxovirus related to equine morbillivirus. *Emerg. Infect. Dis*. **2**, 239–40. 10.3201/eid0203.960315 PMC2626799 8903239

[50] Plowright RK (2011). Urban habituation, ecological connectivity and epidemic dampening: the emergence of Hendra virus from flying foxes (Pteropus spp.). Proc. Biol. Sci..

[51] Field HE (2016). Landscape utilisation, animal behaviour and hendra virus risk. Ecohealth.

[52] Hayman DTS (2013). Ecology of zoonotic infectious diseases in bats: current knowledge and future directions. Zoonoses Public Health.

[53] Hudson, I. L., Kim, S. W. & Keatley, M. R. In Phenological research (eds. Hudson, I. L. & Keatley, M. R.) 209–228 (Springer, 2009).

[54] Giles JR, Plowright RK, Eby P, Peel AJ, McCallum H (2016). Models of Eucalypt phenology predict bat population flux. Ecol. Evol..

[55] Martin GA (2016). Climatic suitability influences species specific abundance patterns of Australian flying foxes and risk of Hendra virus spillover. One Heal..

[56] Markus N, Hall L (2004). Foraging behaviour of the black flying-fox (Pteropus alecto) in the urban landscape of Brisbane, Queensland. Wildl. Res..

[57] Degeling C, Kerridge I (2013). Hendra in the news: Public policy meets public morality in times of zoonotic uncertainty. Soc. Sci. Med..

[58] Wiethoelter, A. *et al*. ‘We’ve learned to live with it’ – A qualitative study of Australian horse owners’ attitudes, perceptions and practices in response to Hendra virus. *Prev. Vet. Med*. (2017). 10.1016/j.prevetmed.2017.03.003 28460752

[59] Edson D (2015). Flying-Fox Roost Disturbance and Hendra Virus Spillover Risk. PLoS One.

[60] Stevens, N., Lehmann, C. E. R., Murphy, B. P. & Durigan, G. Savanna woody encroachment is widespread across three continents. *Glob. Chang. Biol*. 235–244, doi:10.1111/gcb.13409 (2016). 10.1111/gcb.13409 27371937

[61] McFarlane R, Becker N, Field H (2011). Investigation of the climatic and environmental context of Hendra virus spillover events 1994–2010. PLoS One.

[62] Smith C, Skelly C, Kung N, Roberts B, Field H (2014). Flying-fox species density–a spatial risk factor for Hendra virus infection in horses in eastern Australia. PLoS One.

[63] Smith, C. S. *et al*. Twenty years of Hendra virus: laboratory submission trends and risk factors for infection in horses. *Epidemiol. Infect*. 1–8 doi:10.1017/S0950268816001400 (2016). 10.1017/S0950268816001400 PMC9150281 27357144

