# Supplementary material for: The impact of human population pressure on flying fox niches and the potential consequences for Hendra virus spillover
Source: Sci Rep. 2017 Aug 15;7:8226. doi: 10.1038/s41598-017-08065-z (PMC5557840; doi:10.1038/s41598-017-08065-z)
Supplement: Supplementary file 1 — Supplementary information [file 41598_2017_8065_MOESM1_ESM.pdf]

Supplementary Information

The impact of human population pressure on flying fox niches and the potential consequences for Hendra virus spillover

Michael G. Walsh[1], Anke Wiethoelter[2], M.A. Haseeb[3,4]

[1]Marie Bashir Institute for Infectious Diseases and Biosecurity, Westmead Institute for Medical Research, University of Sydney, Westmead, New South Wales, Australia, [2]Faculty of Veterinary and Agricultural Sciences, University of Melbourne, Melbourne, Victoria, Australia, [3]Department of Epidemiology and Biostatistics, School of Public Health, [4]Departments of Cell Biology, Pathology and Medicine, College of Medicine, State University of New York, Downstate Medical Center, Brooklyn, New York, U.S.A.

Supplemental Table. Rasters used as landscape factors in the ecological niche modeling and point process modeling.

Landscape raster	Spatial resolution	Reference	url
Mean temp during the warmest quarter	30 arc-second	[17]	http://www.worldclim.org/
Mean temp during the warmest quarter	30 arc-second	[17]	http://www.worldclim.org/
Mean precipitation during the wettest quarter	30 arc-second	[17]	http://www.worldclim.org/
Mean precipitation during the wettest quarter	30 arc-second	[17]	http://www.worldclim.org/
Altitude	30 arc-second	[17]	http://www.worldclim.org/
Net human migration 1970 – 1980	30 arc-second	[22]	http://sedac.ciesin.columbia.edu/data/set/popdynamics-global-est-net-migration-grids-1970-2000
Net human migration 1980 – 1990	30 arc-second	[22]	http://sedac.ciesin.columbia.edu/data/set/popdynamics-global-est-net-migration-grids-1970-2000
Net human migration 1990 – 2000	30 arc-second	[22]	http://sedac.ciesin.columbia.edu/data/set/popdynamics-global-est-net-migration-grids-1970-2000
Human footprint	30 arc-second	[23]	http://sedac.ciesin.columbia.edu/data/set/wildareas-v2-human-footprint-geographic
Maximum Green Vegetation Fraction	30 arc-second	[27]	http://landcover.usgs.gov/green_veg.php
MODIS Land Cover Type (MCD12Q1)	15 arc-second	[28]	https://landcover.usgs.gov/global_climatology.php
